# Radial Artery Pseudoaneurysm Following Transradial Catheterization: Recognition, Management, and Prevention

**DOI:** 10.1016/j.jscai.2026.105401

**Published:** 2026-06-11

**Authors:** Michelle C. Carvajal, Shivani Shirodkar, Jessica B. Briscoe, Hilkiah Suga, Ifeanyi D. Chinedozi, Ralf Martz Sulague, Nikhita Athipathy, Nivedita Kumar, AlleaBelle Bradshaw, Hiba Juboori, Ahmet Kilic, Jennifer S. Lawton

**Affiliations:** a Division of Cardiac Surgery, Department of Surgery, Johns Hopkins University, Baltimore, Maryland; b Department of Surgery, Cedars-Sinai Medical Center, Los Angeles, California; c Vascular Imaging, Division of Vascular Surgery, Johns Hopkins University, Baltimore, Maryland

**Keywords:** percutaneous coronary intervention, radial artery pseudoaneurysm, transradial catheterization, ultrasound-guided thrombin injection

## Abstract

Transradial cardiac catheterization is recommended over femoral artery catheterization for patients with acute coronary syndrome due to lower rates of bleeding and vascular complications. Despite its favorable safety profile, radial artery-specific complications occur, including spasm, occlusion, arteriovenous fistula, perforation, and pseudoaneurysm. Radial artery pseudoaneurysm (RAP) is an uncommon but potentially morbid access-site complication that remains incompletely characterized, with limited practical guidance on diagnosis and management. This review synthesizes published clinical experience on RAP following transradial catheterization, with key considerations for diagnosis, risk stratification, and management. Across published experience, patients with RAP most commonly present with localized pain, swelling, or a pulsatile mass at the access site, with symptom onset ranging from hours to weeks after catheterization. Duplex ultrasonography is the primary diagnostic modality, allowing assessment of pseudoaneurysm size, neck morphology, and flow characteristics. Management strategies range from observation and mechanical compression to ultrasound-guided thrombin injection and surgical repair, with treatment selection guided by lesion size, symptoms, stability, anticoagulation status, and the need to preserve the radial artery. Early recognition with ultrasound and individualized treatment can prevent progression and associated morbidity as transradial access continues to expand.

## Introduction

More than one million coronary artery catheterizations are performed across the United States per year.^1^ A retrospective analysis from the Veterans Affairs health care system between 2011 and 2018 demonstrated that diagnostic transradial catheterization (TRC) increased from approximately 18% to 60% during this time frame. However, other studies have found much lower adoption rates.^1^ Compared with the transfemoral approach, randomized controlled trials have shown that TRC is associated with significantly lower rates of bleeding, all-cause vascular complications, and mortality in select high-risk patients with acute coronary syndrome.^2^^,^^3^ These studies have also reported higher patient satisfaction and quality of life with the use of transradial versus femoral access.

Although TRC is generally regarded to be safe, vascular complications do occur as a result of radial access.^4^, ^5^, ^6^, ^7^, ^8^, ^9^ Radial artery spasm and occlusion are most prevalent, followed by radial arteriovenous fistula, perforation, and pseudoaneurysm.^4^^,^^7^^,^^8^ TRC for coronary angiography has a class I recommendation in patients with acute coronary syndrome in the 2021 American College of Cardiology/American Heart Association/Society for Cardiovascular Angiography & Interventions Guideline for Coronary Artery Revascularization.^10^ Despite this widespread adoption, less common access-site complications such as radial artery pseudoaneurysm (RAP) remain incompletely characterized, and practical guidance for recognition and management is limited.

As transradial access continues to expand, familiarity with the presentation, diagnostic evaluation, and management of RAP is increasingly important for interventional and vascular specialists. To contextualize available evidence, we summarize key clinical characteristics, management strategies, and outcomes reported in published cases of RAP following TRC, with key considerations for diagnosis, risk stratification, management, and prevention of this rare but potentially morbid complication.

## Clinical presentation, diagnosis, and risk stratification

Patients with RAP after coronary angiography typically present with a pulsatile mass with or without an audible bruit on auscultation (Table 1^5^^,^^11^, ^12^, ^13^, ^14^, ^15^, ^16^, ^17^, ^18^, ^19^, ^20^, ^21^, ^22^, ^23^, ^24^, ^25^, ^26^, ^27^, ^28^, ^29^, ^30^, ^31^, ^32^, ^33^, ^34^, ^35^, ^36^, ^37^, ^38^, ^39^, ^40^, ^41^, ^42^, ^43^, ^44^, ^45^, ^46^, ^47^, ^48^, ^49^, ^50^, ^51^ and Supplemental Table S1). The enlarging mass may elicit pain or compression neuropathy.^27^ Diagnosis is confirmed using duplex ultrasonography with the finding of flow within a pulsatile mass. Color Doppler ultrasound is the initial modality of choice due to its noninvasive nature, accessibility, and capacity to delineate vascular structures and flow dynamics. However, other diagnostic techniques include contrast-enhanced computed tomography angiography, which offers precise arterial anatomy visualization crucial for any intervention planning. Magnetic resonance angiography is advantageous for its avoidance of ionizing radiation and use in patients with iodinated contrast contraindications. Digital subtraction angiography, the gold standard for vascular imaging, is reserved for scenarios necessitating endovascular treatment or when alternative modalities yield inconclusive results. This modality can evaluate the size and anatomy of the pseudoaneurysm with precision.

**Table 1:** Summary of published clinical experience with radial artery pseudoaneurysm following transradial catheterization

Author(s) (Year)	Mean age, y	%, Sample size	Comorbidity	Time from catheterization to symptoms	Chief complaints/signs	Anticoagulation and antiplatelet
Williams and Eccleshall (2009)^11^	79	0, (n =1)	NR	5 d	Painful swelling	NR
Collins et al (2012)^12^	62.2	20, (n = 5)	VHD (2/5), CAD (2/5), DM (1/5), HTN (1/5), Obesity (2/5), OSA (1/5)	2 wk, 2 mo, 1 mo, 1 wk, 1 d, respectively	Painful swelling onsite (1,4); painless swelling onsite (2,3,5)	Yes in 4/5: heparin (1/5), warfarin (1/5), enoxaparin (2/5)
Hamid et al (2012)^13^	81.5	0, (n =2)	DM (1/2), HTN (1/2), VHD (1/2), CAD (2/2), Afib (1/2)	1 d, 2 d	Painless, pulsatile mass + painful swelling of right forearm (1); pulsatile swelling (2)	ASA, clopidogrel, heparin
Nazer and Boyle (2013)^14^	77	0, (n =1)	Rheumatic heart disease	4 d and recurred after 3 wk	Painful, pulsatile mass	Heparin
Cauchi et al (2014)^15^	45	100, (n =1)	NR	Immediate post-op	Painful swelling	ASA, prasugrel
Kleczynski et al (2014)^16^	70	NR, (n= 1)	NR	NR	NR	NR
Zegri et al (2015)^17^	76	80, (n =5)	None	4-17 d	Pulsatile mass	Warfarin (4/5)
Korabathina et al (2015)^18^	56	100, (n = 1)	DM, CAD	5 mo	Swelling	ASA, prasugrel, bivalirudin
Mohamed et al (2015)^19^	85	0, (n = 1)	CAD, HTN, Hypothyroidism	2 h	Pulsatile mass	NR
Samaranayake et al (2015)^20^	85	0, (n = 1)	CAD	1 d	Painless pulsatile mass	Clopidogrel, ASA, enoxaparin, heparin
Tatli et al (2015)^5^	57	0, (n = 1)	HTN, CAD	13 d	NR	NR
Barış et al (2016)^21^	73	0, (n = 1)	Afib, CAD	10 d	Painful swelling	Warfarin, heparin
Charfeddine (2016)^22^	64	0, (n = 1)	HTN, VHD, CAD	7 mo	Painful swelling	Warfarin, heparin, acenocoumarin (stopped 3 d before catheterization)
Babunashvili et al (2017)^23^	68	100, (n = 1)	Afib, CAD, CHF, HTN	2 d	Painful pulsatile Mass	Warfarin, clopidogrel
Ghanavati et al (2017)^24^	32	100, (n = 1)	CAD	2 mo	Painless pulsatile mass	ASA, clopidogrel, warfarin
Sinha et al (2017)^25^	43	0, (n = 1)	CAD, HTN, dyslipidemia	1 d	Swelling	Heparin, aspirin, prasugrel
Tosti et al (2017)^26^	70	18, (n = 11)	NR	9 d	Bleeding (1/11); fever (1/11); pain (4/11); NR (5/11)	ASA (7/11), warfarin (3/11), clopidogrel (3/11), enoxaparin (2/11), heparin (1/11), fondaparinux (1/11), rivaroxaban (1/11)
Alerhand et al (2018)^27^	57	0, (n = 1)	Afib, CAD	5 d	Painful swelling, paresthesia	Apixaban
Kongunattan and Ganesh (2018)^28^	70	0, (n = 1)	DM, CAD	1 d	Swelling, ecchymosis	ASA, clopidogrel, heparin
Tsiafoutis et al (2018)^29^	65	100, (n = 1)	Afib, HTN	7 d	Pulsatile mass	NR
Pathak et al (2018)^30^	64	0, (n = 1)	CAD, HTN	NR	Painful swelling	Heparin, ASA, clopidogrel
Boumezrag et al (2019)^31^	NR	100, (n = 1)	CAD, VHD	Immediate	Pain	Warfarin, enoxaparin
Gallinoro et al (2019)^32^	84	0, (n = 1)	Afib, HTN, cancer	4 d	Painful Hematoma	ASA, Clopidogrel, Enoxaparin
Iftikhar et al (2019)^51^	82	0, (n = 1)	Afib, CAD, HTN	1 d	Pulsatile mass	NR
Mizuguchi et al (2019)^33^	77	0, (n = 1)	HOCM	1 d	Hematoma	NR
Palaparti et al (2019)^34^	69.2	25, (n = 4)	HTN (1/4), DM (1/4), hypothyroid (1/4); CAD (2/4)	7 d (2/4); 2 d (1/4); 3 d (1/4)	Painless mass (2/4); swelling (2/4)	ASA (4/4), ticagrelor (3/4), clopidogrel (1/4), heparin (4/4)
Prejean et al (2019)^35^	75	0, (n = 1)	HTN, HLD, PAD, CAD	2 h	Painful pulsatile mass	Heparin, ASA, prasugrel
Wu L et al (2019)^36^	75	0, (n = 1)	Afib, CAD, ESRD	2 d	Swelling	Warfarin, ASA, clopidogrel
Blanco et al (2020)^37^	93	0, (n = 1)	VHD, cancer	NR	Painful swelling	ASA
Kiat et al (2020)^38^	76	0, (n = 1)	HTN, DM, HLD	8 d	Painful swelling	ASA, clopidogrel, heparin
Tsiafoutis et al (2020)^39^	82	100, (n = 1)	NR	NR	NR	NR
Molina-Lopez et al (2021)^40^	82	100, (n = 1)	Afib, CAD, DM, CKD, HTN	2 d	Painless mass	Ticagrelor, heparin, bivalirudin, clopidogrel, apixaban
Nykl et al (2021)^41^	60	0, (n = 1)	Afib, pulmonary HTN, HTN, HLD, OSA	72 d	Pulsatile painful mass	Apixaban, intra-arterial heparin
Oliveira et al (2021)^42^	75	0, (n = 1)	HTN, hypothyroidism, DM, CAD	6 d	Painful mass, ecchymosis	NR
Prakash et al (2021)^43^	82	100, (n = 1)	CAD	2 d	Swelling, ecchymosis	ASA, clopidogrel, heparin, apixaban
Sharma et al (2021)^44^	77	0, (n = 1)	Afib, CHD, CAD	4 mo	Painful mass	Apixaban
Berrio-Caicedo et al (2022)^45^	74	100, (n = 1)	Afib, HTN	Immediate	Pain, paresthesia	Heparin started 5 d prior to procedure; at time of procedure, heparin stopped and DOAC started
Bolt et al (2022)^46^	42	100, (n = 1)	VHD	NR	Palpable thrill	NR
Campbell et al (2022)^47^	74	100, (n = 1)	CHF, VHD, Afib	4 wk	Painful swelling	Rivaroxaban
Maeba et al (2022)^48^	71	100, (n = 1)	CAD	3 mo	Pain, paresthesia	NR
Papadoulas et al (2022)^49^	83	100, (n = 1)	CAD, HTN, HLD	1 wk	Painful pulsatile mass	NR
Pinxterhuis, T. (2022)^50^	71	100, (n = 1)	NR	Immediate post-op	Painful swelling	NR

Published reports of radial artery pseudoaneurysm after transradial catheterization and associated clinical features and antithrombotic therapy. Data were extracted from published reports.

Afib, atrial fibrillation; ASA, acetylsalicylic acid; CAD, coronary artery disease; CKD, chronic kidney disease; DM, diabetes mellitus; DOAC, direct oral anticoagulants; ESRD, end-stage renal disease; HLD, hyperlipidemia; HOCM, hypertrophic obstructive cardiomyopathy; HTN, hypertension, NR, not reported; OSA, obstructive sleep apnea; PAD, peripheral artery disease; VHD, valvular heart disease.

The time from TRC to onset of symptoms may range from hours to several years. Chadow et al reported a case of a patient with thickened intima and dissection of the radial artery identified 8 years after transradial access.^52^ Clarke et al also reported a patient with chronic dissection, thickened intima, and near lumen occlusion after harvesting the radial artery 12 years following radial artery cannulation, illustrating the long-term structural consequences of prior radial access even in the absence of a discrete pseudoaneurysm.^53^

Although the radial artery is favored for percutaneous coronary intervention because of lower bleeding and vascular complication rates compared with femoral access, at least one case report has raised concern that prior TRC may be associated with reduced graft patency when the artery is later harvested for coronary artery bypass grafting, although broader evidence on this association remains limited.^54^ These observations underscore the importance of strategic radial artery use, including limiting repeat access and preserving at least one radial artery when surgical revascularization may be anticipated.

Published reports describing RAP following TRC are summarized in Table 1, highlighting patient characteristics, clinical presentation, diagnostic modalities, management strategies, and reported outcomes.^5^^,^^7^^,^^11^, ^12^, ^13^, ^14^, ^15^, ^16^, ^17^, ^18^, ^19^, ^20^, ^21^, ^22^^,^^24^, ^25^, ^26^, ^27^, ^28^, ^29^, ^30^, ^31^, ^32^, ^33^, ^34^, ^35^, ^36^, ^37^, ^38^, ^39^, ^40^, ^41^, ^42^, ^43^, ^44^, ^45^, ^46^, ^47^, ^48^, ^49^^,^^51^^,^^55^

## Management of RAP

Management of RAP follows a pragmatic, stepwise approach guided by lesion size, symptoms, anatomic features, anticoagulation status, and the need to preserve radial artery patency (Central Illustration).

**Central Illustration:**
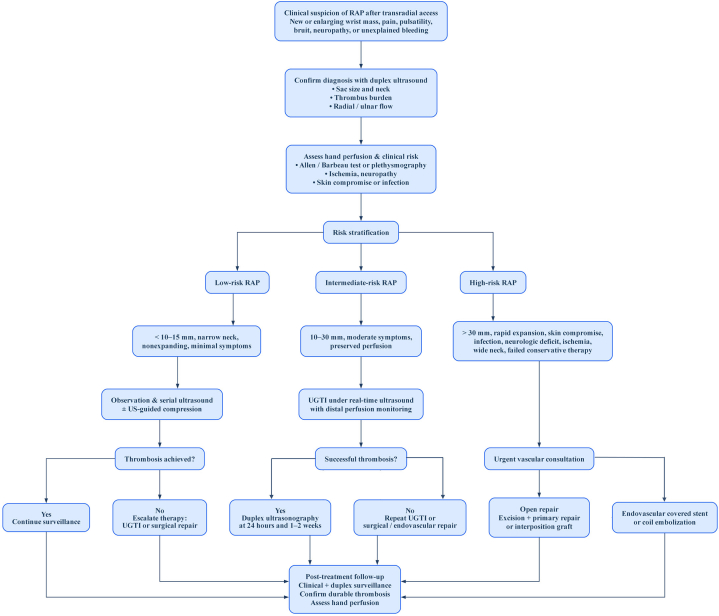
**Proposed risk-stratified management algorithm for radial artery pseudoaneurysm (RAP) after transradial access, incorporating pseudoaneurysm size, symptoms, perfusion status, and response to initial therapy.** RAP, radial artery pseudoaneurysm; UGTI, ultrasound-guided thrombin injection; US, ultrasound.

### Conservative management and mechanical compression

Observation with planned serial duplex is reasonable for stable RAP <3 cm in patients without significant pain, neurological symptoms, or skin changes. In a series of 16 RAPs, lesions smaller than 3 cm were managed nonoperatively with monitoring and serial ultrasound demonstrated spontaneous thrombosis within approximately 3 weeks.^17^^,^^26^

Mechanical compression, either manual or device-based, represents the next step for suitable lesions. Ultrasound-guided manual compression allows targeted obliteration of RAP neck flow while monitoring radial and ulnar perfusion.^56^ Experience from femoral pseudoaneurysms shows ultrasound-guided compression success rates in the range of approximately 75% to 90%, with lower efficacy in anticoagulated patients and in larger pseudoaneurysm sacs.^56^, ^57^, ^58^

Radial compression bands originally designed for postprocedural hemostasis have been repurposed therapeutically. Differential compression techniques combining proximal radial artery compression with intermittent ulnar artery occlusion have been described to reduce inflow and promote thrombosis.^34^^,^^59^ These approaches may be particularly useful in frail patients or those at high surgical risk, provided that hand perfusion is maintained and close clinical and sonographic surveillance is ensured.

Molina-López et al described an attempt at RAP thrombosis using a transradial compression band to occlude the radial artery proximally, combined with intermittent ulnar artery occlusion to dampen retrograde flow, with intermittent assessment using point-of-care ultrasound. Although thrombosis was not achieved, this approach represents a potential noninvasive strategy that may warrant further exploration.^40^

### Ultrasound-guided thrombin injection

Ultrasound-guided thrombin injection (UGTI) has become a cornerstone of pseudoaneurysm management across vascular territories and is increasingly applied to the radial artery.^16^^,^^60^ Extrapolating from large femoral series and mixed pseudoaneurysm cohorts, UGTI offers high success rates with low complication risk when performed with meticulous technique. In a prospective series of 240 femoral and brachial artery postcatheterization pseudoaneurysms, primary success rates reached 94%, with only 2 transient thromboembolic events.^60^

Radial-specific data remain limited but favorable. Komorowska-Timek et al reported treatment of radial and ulnar pseudoaneurysms using percutaneous thrombin injection, with durable thrombosis and preserved distal perfusion.^61^ Garvin et al described safe UGTI for upper extremity pseudoaneurysms in anticoagulated patients.^62^ More recent case reports document successful treatment of RAP, including lesions arising after distal radial access, using low-dose thrombin (typically 100-500 IU) injected under real-time ultrasound guidance.^63^, ^64^, ^65^

Anatomical considerations are critical, as RAP with a short, wide neck, minimal separation between the sac and parent artery, and proximity to major branch points are generally poor candidates for UGTI because of the risk of thrombin entering the native artery and causing nontarget thrombosis.^16^^,^^40^^,^^60^, ^61^, ^62^, ^63^, ^64^, ^65^, ^66^ Unlike femoral artery pseudoaneurysm management, for which formal neck-size thresholds are well-established to guide UGTI candidacy, analogous RAP criteria have not yet been prospectively validated. Given the smaller caliber of the radial artery and the proximity of adjacent structures, neck dimensions may carry even greater clinical significance in this territory, and prospective studies incorporating standardized neck measurements would help clarify whether formal neck-based criteria can improve the safety and success of UGTI for RAP.

### Surgical repair

Open repair remains the definitive therapy for large, expanding, infected, or complicated RAP and for lesions that have failed conservative, compression, or UGTI approaches. Surgery is mandatory in the presence of skin necrosis, compartment syndrome, rapidly enlarging hematoma, or distal ischemia, and is often preferred in patients with compressive neuropathy or concomitant arteriovenous fistula.^25^^,^^49^

Operative strategies include pseudoaneurysm excision with primary arterial repair, patch angioplasty, or interposition grafting, depending on the extent of arterial injury and conduit quality. In most series, primary repair is feasible for wrist-level RAP and is associated with excellent limb outcomes. Arterial ligation is reserved for situations in which reconstruction is not possible and the ulnar circulation is clearly adequate but sacrifices the radial artery as a future conduit.^7^^,^^25^^,^^45^^,^^48^^,^^49^

### Endovascular and hybrid approaches

Endovascular options, particularly covered stent implantation and coil embolization, provide useful alternatives for anatomically suitable RAPs, especially in patients at high surgical risk or with hostile local anatomy. Covered stents have been used to exclude the pseudoaneurysm neck while maintaining radial patency, including via distal radial access, though it should be noted that the small caliber of the radial artery may predispose to stent restenosis and occlusion, as has been observed with covered stents in small coronary vessels.^29^^,^^67^

Coil embolization of the affected radial segment, with or without proximal balloon occlusion, is an established strategy when radial sacrifice is acceptable and ulnar perfusion is robust. Case reports describe durable thrombosis and preserved hand perfusion following embolization of upper extremity pseudoaneurysms.^68^

### Prevention of RAP

RAP prevention parallels best practices for minimizing radial injury and occlusion, particularly in patients who may require future coronary artery bypass grafting. Advanced age, female sex, hypertension, small or calcified radial arteries, and intensive antithrombotic therapy increase access-site bleeding and pseudoaneurysm risk, warranting fewer puncture attempts, gentle wire/catheter handling, and a low threshold for alternative access.^4^^,^^69^^,^^70^

Preservation of at least one radial artery for grafting should be built into procedural planning in patients with multivessel coronary disease, favoring femoral when surgical revascularization is anticipated. Ultrasound-guided puncture, optimized sheath-to-artery ratio, adequate intraprocedural anticoagulation, and prompt angiography when resistance or pain suggests perforation or dissection reduce arterial wall trauma that predisposes to RAP.^5^^,^^7^^,^^9^

Hemostasis should be achieved while preserving radial artery flow: device placement directly over the puncture, early graded deflation to maintain antegrade flow, and avoidance of both under- and overcompression.^4^^,^^7^, ^8^, ^9^ Finally, routine education on access-site self-inspection should be offered, plus brief structured follow-up to facilitate early recognition and noninvasive management of evolving RAP.

## Risk factors for RAP development

RAP formation following TRC has been associated with multiple factors, including inappropriately sized vascular sheaths, failure of arterial closure device or mechanical compression, excessive postprocedural anticoagulation,^69^ and patient-related characteristics such as advanced age and female sex.^70^ The reported incidence of RAP following catheterization varies widely in the literature and ranges from 0.01% to 0.20%.^4^^,^^5^ Clinically, patients may be asymptomatic or present with pain, swelling, and a pulsatile mass at the catheterization site, with symptom onset occurring within hours of the procedure or delayed by several weeks.^11^^,^^12^

Published clinical experience suggests that RAP following coronary angiography is more frequently reported in older patients, women, those with hypertension, and individuals undergoing interventional coronary procedures or receiving anticoagulant or antiplatelet therapy.^69^^,^^70^ A single-center study by Delf et al found a higher incidence of peripheral pseudoaneurysms among women (62%) with a median age of 72 years.^70^ Anticoagulation may influence pseudoaneurysm size and response to conservative therapy, although no large comparative studies have confirmed this association.^69^

Hypertension is commonly reported among affected patients and may be a risk factor for pseudoaneurysm due to its known association with pathologic vasoconstriction, impaired vasodilation, and compromise of the integrity of the arterial wall, likely predisposing patients to vascular complications after radial artery catheterization.^55^

Furthermore, published reports suggest a higher risk of RAP and other vascular complications during interventional versus diagnostic procedures. Procedure-related factors such as sheath size, duration, degree of manipulation, and number of puncture attempts may contribute to this increased risk.^4^^,^^5^^,^^9^

### Limitations

Limitations include that the available literature on RAP is composed predominantly of single case reports and very small series, with no prospective registries or randomized studies to date. This inherently limits the strength of any conclusions drawn regarding incidence, optimal treatment thresholds, or comparative efficacy of management strategies. These limitations underscore the need for prospective, multicenter data collection to better characterize the natural history and management of this rare complication. Nevertheless, given the rarity of RAP, large prospective studies remain difficult to conduct, and this synthesis of published cases offers practical guidance for clinicians managing this uncommon complication.

## Conclusion

Patients with RAP typically present with localized, often painful or pulsatile wrist swelling detectable by duplex ultrasonography. Early recognition and risk stratification are essential, as management ranges from observation and compression to thrombin injection or surgical repair depending on lesion characteristics, symptoms, and stability. Sonography remains the preferred modality to characterize pseudoaneurysm anatomy and guide treatment decisions. This review highlights the importance of vigilant postcatheterization assessment and a pragmatic, individualized management approach to enable timely intervention, minimize complications, and preserve radial artery integrity as transradial access continues to expand.

## CRediT authorship contribution statement

**Michelle C. Carvajal:** Writing – review & editing, Writing – original draft, Formal analysis, Data curation, Conceptualization. **Shivani Shirodkar:** Writing – review & editing, Formal analysis, Data curation. **Jessica B. Briscoe:** Formal analysis, Data curation, Conceptualization. **Hilkiah Suga:** Writing – review & editing, Formal analysis, Data curation. **Ifeanyi D. Chinedozi:** Writing – review & editing, Formal analysis, Data curation, Conceptualization. **Ralf Martz Sulague:** Formal analysis, Data curation. **Nikhita Athipathy:** Formal analysis, Data curation. **Nivedita Kumar:** Formal analysis, Data curation. **AlleaBelle Bradshaw:** Writing – review & editing, Formal analysis, Data curation, Conceptualization. **Hiba Juboori:** Formal analysis, Data curation. **Ahmet Kilic:** Writing – review & editing, Data curation. **Jennifer S. Lawton:** Writing – review & editing, Writing – original draft, Supervision, Resources, Project administration, Formal analysis, Conceptualization.

## Declaration of competing interest

The authors declared no potential conflicts of interest with respect to the research, authorship, and/or publication of this article.

## Footnotes

To access the supplemental material accompanying this article, visit the online version of the *Journal of the Society for Cardiovascular Angiography & Interventions* at 10.1016/j.jscai.2026.105401.
