# Supplementary material for: Radial Artery Pseudoaneurysm Following Transradial Catheterization: Recognition, Management, and Prevention
Source: J Soc Cardiovasc Angiogr Interv. 2026 Jun 11;5(7):105401. doi: 10.1016/j.jscai.2026.105401 (PMC13400115; doi:10.1016/j.jscai.2026.105401)
Supplement: Supplementary Table S1 [file mmc1.docx]

**SUPPLEMENTAL MATERIALS**

**Table S1: Reported management strategies and outcomes in published cases of radial artery pseudoaneurysm.**

| First Author (Year) | Study Type | Country | Incidence | Mean Size of PSA Non-op (cm) | Mean Size of PSA-Op (cm) | Surgical Repair | Non-surgical Treatment Used | Time to Recovery | Outcome |
| --- | --- | --- | --- | --- | --- | --- | --- | --- | --- |
| Williams, P. (2009) | Case Report | UK | NR | NR | NR | Yes | N/A | NR | FR |
| Collins, N. (2012) | Case Series | Australia | NR | NA | 1.7x1.0 | Surgical Repair (4/5); Conservative (1/5) | Compressive Clamp Application | NR | NR |
| Hamid, T. (2012) | Case Series | UK | NR | N/A | 2.97 x  1.64 x 2.36/NR | Yes (2/2) | No | 10 days (1/2), NR (1/2) | FR (1/2), NR (1/2) |
| Nazer, B. (2013) | Case Report | USA | NR | 1.4 x 0.7 x 1.7 | NR | No | Manual Compression; HemoBand Compression for recurrence | 7 days | FR |
| Cauchi, M. (2014) | Case Report | USA | NR | 1.1 x 0.29 | N/A | No | Compression with TR Band | 1 day | FR |
| Kleczynski, P. (2014) | Retrospective Cohort | Poland | NR | NR | NR | No | Thrombin Injection | NR | FR |
| Zegri, I. (2015) | Case Series | Spain | 0.03% | NR | NR | No | Thrombin (2/5); Compression (2/5); NR (1/5) | NR | FR |
| Korabathina, R. (2015) | Case Report | USA | NR | 2.05 x 0.92 | N/A | No | Radial Compression Band | 1 day | FR |
| Mohamed, M. (2015) | Case Report | USA | NR | 2.5 x 2.3 | N/A | No | Thrombin | 2 weeks | FR |
| Samaranayake, C. (2015) | Case Report | New Zealand | NR | N/A | 3.7 x 2.6 x 1.3 | Yes | N/A | 2 days | NR |
| Tatli, E. (2015) | Cross-sectional | Turkey | 0.0097% | NR | NR | Yes | N/A | NR | NR |
| Barış VÖ. (2016) | Case Report | Turkey | NR | NR | 1.7 x 1.0 | Yes | N/A | 10 days | FR |
| Charfeddine, S. (2016) | Case Report | Tunisia | NR | NR | NR | Yes | N/A | NR | FR |
| Babunashvili, A. (2017) | Case Report | Russia | 0.02% | 4.9 x 3.8 | N/A | No | Sheath kept in lumen for 8 hours + Manual compression | NR | FR |
| Ghanavati, R. (2017) | Case Report | Iran | NR | 2.5 x 2.0 | N/A | No | Compression with TR Band | 1 day | FR |
| Sinha, S. (2017) | Case Report | India | NR | NA | 1.1 x 0.34 | Yes | N/A | NR | FR |
| Tosti, R. (2017) | Case Series | USA | NR | <3 | >3 | Yes (7/11), No (4/11) | NR | 5 to 42 days | FR (10/11); non-op patient death (1/11) |
| Alerhand S. (2018) | Case Report | USA | NR | NR | NR | Yes |  | NR | FR |
| Kongunattan, V. (2018) | Case Report | India | NR | NR | N/A | No | Ultrasound guided compression | 3 days | FR |
| Tsiafoutis, I. (2018) | Case Report | Greece | NR | 2.7 x 1.7 | N/A | No | PCI - Covered Stent | 1 day | FR |
| Pathak, L. (2018) | Case Report | India | NR | 2 PSA:  1 x 0.46 and 1 x 0.69 | N/A | No | Compression with the TR Band | 1 week | NR |
| Boumezrag, M. (2019) | Case Report | USA | NR | NR | NR | No | Embolization | 2 days | FR |
| Gallinoro, E. (2019) | Case Report | Italy | NR | NR | 2.0 x 2.0 | Yes | N/A | 7 days | FR |
| Iftikhara, S (2019) | Case Report | USA | NR | 2.3 x 2.4 x 1.4 | NR | No | Pneumatic band compression | 10 hours | FR |
| Mizuguchi, Y. (2019) | Case Report | Japan | NR | NR | NR | No | Ultrasound guided compression, then hemostatic pad with elastic bandage compression | 7 days | FR |
| Palaparti, R. (2019) | Case series | India | NR | 6 x 8 and 1.1 x 0.7 and NR | 3.8 x 1.8 | No (3/4); Yes (1/4) | Ultrasound guided compression | NR | FR |
| Prejean, S. (2019) | Case report | USA | NR | 2.3 x 1.5 x 1.7 | N/A | No | Compression with the TR Band | 12 hours | FR |
| Wu, L. (2019) | Case report | USA | NR | 2 PSA:  2.05 x 0.68 and 0.7 x 0.6 | N/A | No | Compression with the TR Band | 2 days; 8 days | FR |
| Blanco, A. (2020) | Case report | USA | NR | N/A | 2.6 x 1.2 x 1.7 | Yes | N/A | NR | FR |
| Kiat, J. (2020) | Case series | Singapore | NR | N/A | 1.7 x 1.9 x 3.2 | Yes | N/A | NR | FR |
| Tsiafoutis, I. (2020) | Case report | Greece | NR | NR | N/A | No | PCI - Stent | NR | NR |
| Molina-Lopez, V. (2021) | Case report | USA | NR | 1.8 x 2 x 1.3 | N/A | No | Manual and TR Band compression | NR | FR |
| Nykl, R. (2021) | Case report | Czech Republic | NR | N/A | 1.2x1.3x1.5 | Yes | N/A | 3 days | FR |
| Oliveira, MD (2021) | Case report | Brazil | NR | NR | NR | No | Ultrasound guided compression and TR Band compression | 4 hours | FR |
| Prakash, B. (2021) | Case report | India | NR | N/A | 3.4 x 2.2 | Yes | N/A | NR | FR |
| Sharma, R. (2021) | Case series | USA | NR | N/A | 3.2 x 1.8 | Yes | N/A | NR | NR |
| Berrio-Caceido, J. (2022) | Case report | Colombia | NR | N/A | NR | Yes | N/A | NR | FR |
| Bolt, R. (2022) | Case report | USA | NR | N/A | 0.59 x 0.53 | Yes | N/A | NR | FR |
| Campbell, S. (2022) | Case report | New Zealand | NR | N/A | 3.3 | Yes | N/A | NR | NR |
| Maeba, T. (2022) | Case report | Japan | NR | N/A | NR | Yes | N/A | NR | FR |
| Papadoulas, S. (2022) | Case report | Greece | NR | N/A | NR | Yes | N/A | 3 months | FR |
| Pnxterhuis, T. (2022) | Case report | The Netherlands | NR | NR | NR | Yes | N/A | NR | NR |

PSA-Non-op: Pseudoaneurysm managed nonoperatively; PSA-Op: Pseudoaneurysm managed operatively; NR: Not reported; N/A: Not Applicable; FR: Full Recovery; TR Band: commercial radial compression device; PCI: Percutaneous catheter intervention

**Legend:** Supplemental Table 1 summarizes in detail the findings from Table 1.
